# Targeting the macrophage immunocheckpoint: a novel insight into solid tumor immunotherapy

**DOI:** 10.1186/s12964-023-01384-x

**Published:** 2024-01-25

**Authors:** Bei Zhou, Yan Yang, Yan Kang, Jingjing Hou, Yun Yang

**Affiliations:** 1 Department of Biochemistry and molecular biology, School of Basic Medical Sciences, Xinxiang Medical University, Xinxiang, Henan 453003 China; 2 Department of Gastrointestinal Surgery, Zhongshan Hospital of Xiamen University, Xiamen, Fujian 361004 China; 3 Institute of Gastrointestinal Oncology, School of Medicine, Xiamen University, Xiamen, Fujian 361004 China

**Keywords:** Macrophage, Solid tumor, Immunotherapy, Immune factors

## Abstract

Tumor immunotherapy, which targets immune checkpoints, presents a promising strategy for the treatment of various cancer types. However, current clinical data indicate challenges in its application to solid tumors. Recent studies have revealed a significant correlation between the degree of immune response in immunotherapy and the tumor microenvironment, particularly with regard to tumor-infiltrating immune cells. Among these immune cells, macrophages, a critical component, are playing an increasingly vital role in tumor immunotherapy. This review focuses on elucidating the role of macrophages within solid tumors and provides an overview of the progress in immunotherapy approaches centered around modulating macrophage responses through various immune factors.

Video Abstract

**Supplementary Information:**

The online version contains supplementary material available at 10.1186/s12964-023-01384-x.

## Introduction

Macrophages are a type of white blood cells in the immune system that play an important role in the body, mainly responsible for phagocytosis and digestion of pathogens, cell debris, and other foreign objects. They also participate in the regulation of inflammatory reactions and tissue repair. Their origin can be traced back to the differentiation and development of hematopoietic stem cells. Tumor associated macrophages (TAMs) typically refer to macrophages in the microenvironment of solid tumors, which account for the largest proportion of myeloid cell infiltration in solid tumors and are closely related to poor prognosis in cancer patients [1]. Relevant studies have demonstrated that TAMs exhibit considerable plasticity, being easily polarized into distinct types in response to environmental factors [2]. Within the tumor microenvironment, TAMs exert certain tumor-promoting effects, including the stimulation of tumor cell proliferation, metastasis, and angiogenesis [3, 4]. It is worth noting that existing research has underscored the efficacy of tumor immunotherapy in emphasizing the immune system’s pivotal role in thwarting tumor progression. This therapeutic approach has evolved into a significant treatment modality following surgery, radiotherapy, and chemotherapy, thereby substantially transforming the landscape of cancer treatment [5]. The normal human immune system is equipped with immune surveillance capabilities. In the presence of tumors within the body, the immune system can recognize and specifically target these “non-self” cells through immune mechanisms to counteract the initiation and progression of cancer. However, it is important to note that many tumors possess the capacity for evolutionary selection to evade the immune system’s responses, which manifest in three distinct phases: elimination, equilibrium, and escape. This process is commonly referred to as “immune editing” [6]. By modifying the internal state of tumor cells, manipulating the tumor microenvironment, and employing other mechanisms, tumors can thwart immune-mediated rejection. This subsequently leads to a weakened immune response against the tumor, ultimately enabling the tumor to evade immune surveillance and progress [7]. This dynamic interplay between tumor cells and immune cells induces a state of metabolic competition within the tumor immune microenvironment. Consequently, the effective supply of nutrients is restricted, and the microenvironment’s cellular pH becomes acidic, hampering the functionality of immune cells. Since the early twenty-first century, a diverse array of anti-tumor immune drugs, including immune checkpoint inhibitors and tumor vaccines, has emerged through clinical studies focused on unraveling the mechanisms of immune escape employed by tumors [8]. In recent years, targeted immunotherapy has gained significant prominence in the realm of cancer treatment. Specific immunotargeting of macrophages and molecules associated with the regulation of macrophage function have garnered widespread attention. Macrophages are integral components of the immune system, and their functions extend beyond immune responses, playing crucial roles in tissue repair, malignancy control, and immune regulation. Studies have indicated that the use of specific immunotargeting techniques allows for the targeted delivery of drugs or therapeutic agents to macrophages, thereby enhancing treatment efficacy and reducing unnecessary side effects [9]. This strategy offers a novel approach to cancer therapy, bringing new hope for patients with advanced and metastatic cancers. Furthermore, the regulation of macrophage function is closely linked to the success of tumor immunotherapy. Various cytokines, receptors, and molecular signaling pathways participate in the activation and suppression of macrophages, thereby influencing the effectiveness of immune responses [10]. Understanding the mechanisms of action of these molecules and how to precisely regulate macrophage function is pivotal for the success of immunotherapy. For instance, by inhibiting inflammatory responses or activating immune checkpoint molecules, we can enhance treatment efficacy, reduce side effects, and ultimately improve patient survival and quality of life. Consequently, tumor immunotherapy has garnered mounting interest among scholars both domestically and internationally.

## Immunosuppression of macrophages and tumor microenvironment

As widely acknowledged, macrophages constitute the principal effector cells in the later stages of the innate immune response. They also partake in antigen presentation within the realm of adaptive immunity. Originating from bone marrow stem cells, monocytes undergo differentiation into mature macrophages within specific tissues. These mature macrophages demonstrate substantial plasticity in their biological attributes. Upon encountering different microenvironments or activation stimuli, such as toll-like receptor (TLR), lipopolysaccharide (LPS), interferon factor-γ (IFN-γ), and TNF-α, macrophages adopt the M1-type activation state, also known as classical activated macrophages. M1-type macrophages are characterized by their secretion of significant quantities of proinflammatory cytokines (IL-6 and IL-1β), inducible nitric oxide synthase (iNOS), and cyclooxygenase 2 (COX2), all of which exert proinflammatory effects and are pivotal in the clearance of invading microorganisms. Conversely, stimulation by IL-4 or IL-13 results in the activation of macrophages into the M2-type, or alternatively activated macrophages. M2-type macrophages are known for their ability to secrete anti-inflammatory cytokines, such as IL-10 and IL-1 receptor antagonists, and they highly express arginase 1 (ARG1) and COX1, which have anti-inflammatory properties. These macrophages are instrumental in facilitating tissue repair and remodeling following damage [11].

**Fig. 1:**
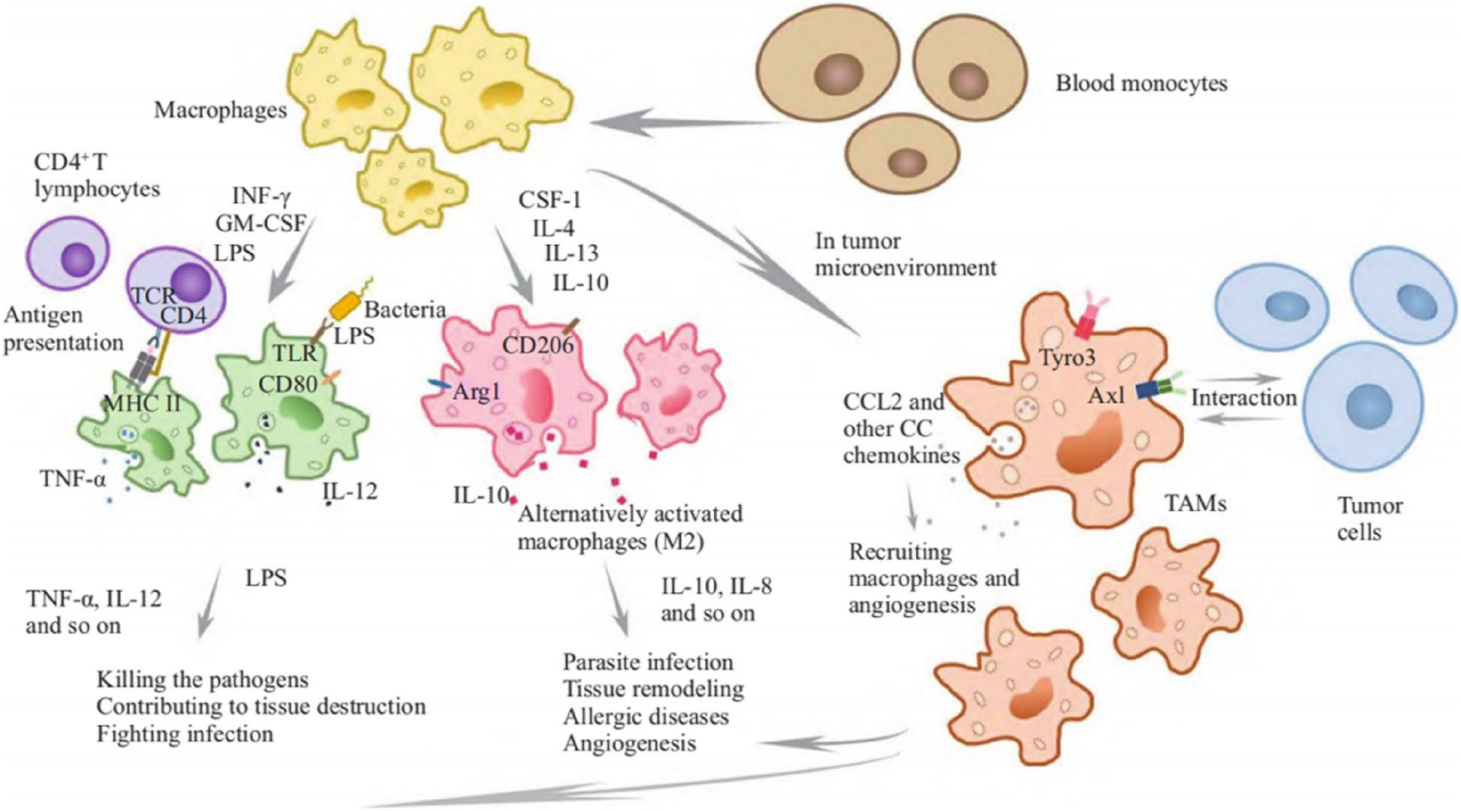
Two polarized subtypes of macrophages (It’s worth noting that macrophage polarization is a dynamic process, allowing macrophages to maintain tissue homeostasis and overall physiological balance in response to changing microenvironments)

It is imperative to acknowledge that macrophages are profoundly influenced by the dynamic microenvironment, rendering their phenotype and function highly heterogeneous. Achieving the clear polarization of macrophages into the ideal M1 or M2 type is indeed challenging [12]. TAMs exhibit a remarkable ability to swiftly adapt to changes within the tumor microenvironment. While functionally similar to M2-type macrophages, TAMs do not align entirely with this classification, instead displaying characteristics that promote microenvironmental immunosuppression and tumor progression [13]. The immunosuppressive role of TAMs is primarily intertwined with the types and functions of infiltrating T cells within the microenvironment. TAMs can directly impede the immune functions of cytotoxic T lymphocytes (CTL) through at least three distinct mechanisms: 1. Expression of immune checkpoints, including programmed cell death 1 ligand 1 (PD-L1) and B7-H4, with subsequent interaction with CTL. This interaction negatively regulates CTL immune function, thereby diminishing its anti-tumor effectiveness. 2. The secretion of immunosuppressive factors such as IL-10 and TGF-β, leading to CTL functional impairment. 3. The regulation of the expression of certain metabolites that can influence CTL activity via metabolic pathways. For instance, TAMs, which overexpress ARG1, lead to the breakdown of L-arginine, a critical component for CTL anti-tumor activity [14]. Furthermore, TAMs can affect CTL immune function by recruiting immunosuppressive cells like Treg cells, limiting the capacity of dendritic cells for antigen presentation, and influencing vascular structures. These actions collectively contribute to the immune escape of tumor cells and the establishment of an immunosuppressive microenvironment [15]. The two polarized macrophage subtypes are illustrated in Fig. 1 and Table 1.

**Table 1:** Immune factors and macrophages

Immune factors	Effects	Biological effects
CD68	Macrophage markers	Used for macrophage identification and recognition
CD163	Macrophage specific receptor, chemokine	Clearing free hemoglobin in the body, maintaining tissue homeostasis
PD-L1	Immune checkpoint molecule	Inhibiting T cell immune responses, regulating immune reactions
CD47	Antigen, which interacts with macrophage receptors	Inhibiting macrophage phagocytosis, assisting cells in evading phagocytosis
CD24-Siglec-10	CD24 and Siglec-10, surface molecules, and immunosuppressive receptors	Siglec-10 interacts with CD24 to inhibit macrophage activity and regulate immune responses

## Effects of various immune factors on macrophages

**Fig. 2:**
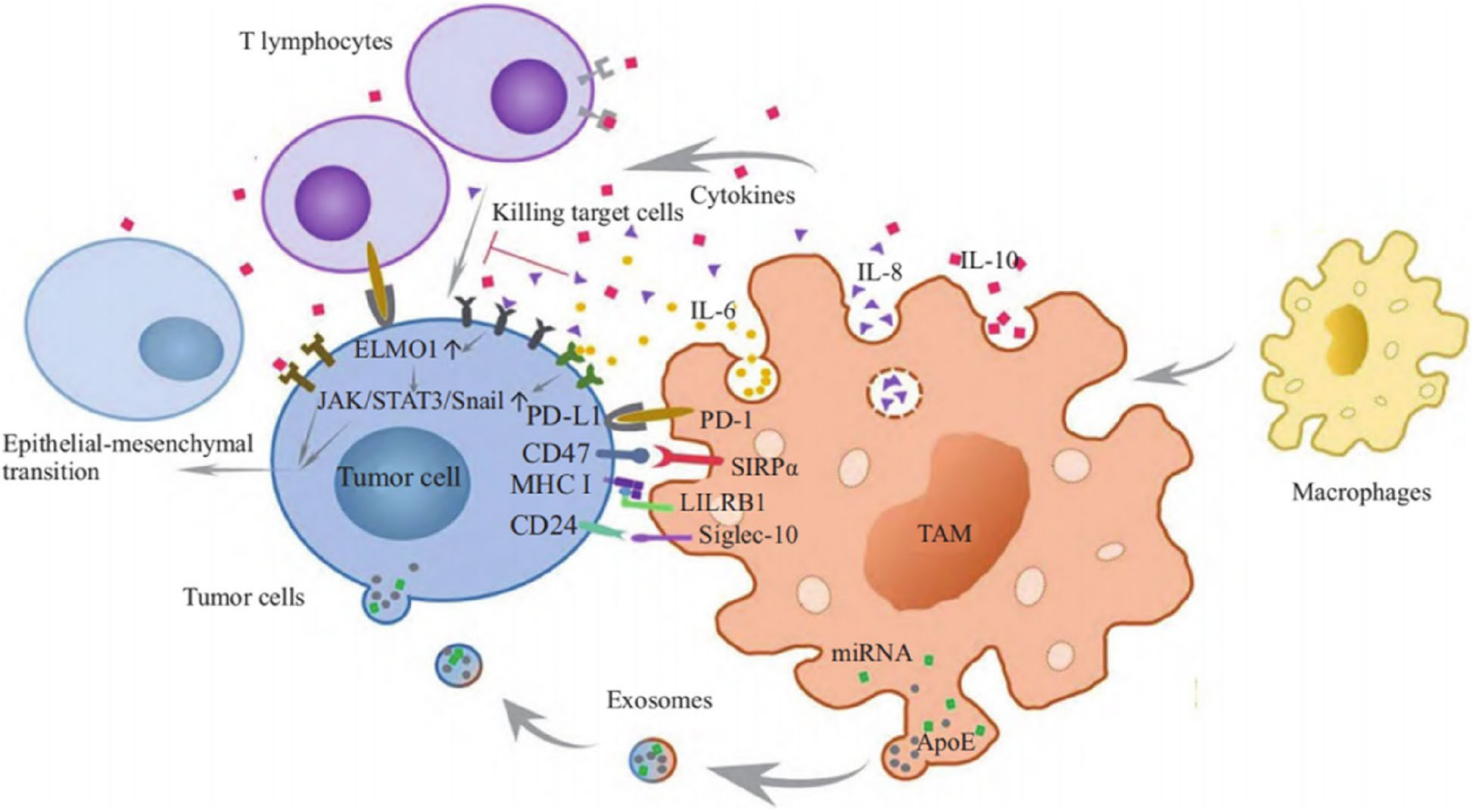
TAMs affect tumor progression through a variety of cytokines and signaling pathways (TAMs can secrete growth factors and chemicals that promote angiogenesis, promoting tumor growth and proliferation. This can include inhibiting T cell activity, hindering immune cell infiltration, and weakening anti-tumor immune responses. Then, it can promote inflammatory response, increase cytokine secretion, and thus affect the microenvironment around tumors)

In the context of immune cells’ specific recognition and elimination of tumor cells, the mechanisms underlying the recognition and clearance of tumor cells are intricate, given the participation of various components of the immune system. Among these components, macrophages stand out as one of the pivotal elements. TAMs are extensively present in the stromal regions of various tumors, playing a significant role in the progression of malignancies and the evasion of immune responses. The examination of the intricate interplay between TAMs and diverse cell types during tumor progression has established a groundwork for innovative approaches to tumor therapy centered around TAMs. While it is widely accepted that TAMs, when present in proximity to malignant tumor cells, can foster tumor proliferation and metastasis, it’s essential to recognize that only specific subtypes of macrophages exhibit anti-tumor activities Fig. 2.

### CD68 and macrophages

TAMs present in the tumor microenvironment primarily originate from peripheral blood mononuclear cells, accumulating in tumor sites under the influence of chemokines. The presence of CD68, recognized as a marker reflecting the overall infiltration of TAMs in tumors, is associated with a tumor-promoting role of CD68 + TAMs in the context of tumor progression. CD68 plays a crucial role in the immune system and inflammatory responses, aiding macrophages in their phagocytic and debris-clearing functions. The expression of CD68 is regulated by various factors, including cytokines and immune modulatory molecules. One of the primary functions of CD68 is its involvement in phagocytosis. It can interact with surface molecules on other cells and secreted substances, thereby assisting macrophages in recognizing, engulfing, and digesting pathogens, dead cells, or other cellular materials. This function is of paramount importance in tumor immune responses and the activity of tumor-associated macrophages. Investigations have revealed that CD68 + TAMs are linked to lymph node metastasis and poor histological grades in breast cancer cases. Additionally, pancreatic cancer patients with a high density of CD68 + TAMs within the tumor stroma tend to exhibit significantly reduced overall survival rates. Subsequent research has unveiled the heterogeneity of CD68 + TAMs, constituting distinct subgroups capable of adapting to various stimuli in the tumor microenvironment, thereby polarizing into different phenotypes [16]. CD68 + TAMs can undergo polarization into M1 and M2 types. The M1 subtype represents the classical activation phenotype, characterized by an enhanced secretion of cytokines like IL-1β, IL-6, and TNF-α, ultimately contributing to an anti-tumor inflammatory response. Conversely, the M2 subtype exhibits an alternative activation phenotype, characterized by an elevated secretion of inflammatory factors, fostering tumor progression [17].

### CD163 and macrophages

CD163 recognizes and binds to free hemoglobin in the bloodstream, initiating signaling pathways, including STAT3, NF-κB, and Akt, among others, to regulate cell survival, proliferation, and differentiation. The activation of these signaling pathways can lead to changes in cellular responses, including cell cycle regulation and gene expression control, thereby influencing tumor development. It’s important to note that the role and significance of CD163 may vary in different types of cancers, and its physiological effects are influenced by the tumor type and individual variations. CD163, recognized as a specific marker for M2-type TAMs, serves the purpose of distinguishing the M2-type TAMs subgroup from the overall TAM population. Several studies have indicated a predilection for TAMs in tumors to exhibit the M2 phenotype (CD163), with a heightened density of M2-type TAMs being linked to unfavorable patient prognosis. In a bid to further elucidate the role of TAMs in tumorigenesis and progression, several investigations have concurrently assessed the infiltration of CD68 + TAMs and CD163 + TAMs within tumors [18]. A study focused on ovarian cancer, for instance, unveiled a significant increase in both CD68 + TAMs and CD163 + TAMs density in advanced ovarian cancer, with CD163 + TAM infiltration being associated with an adverse prognosis in ovarian cancer patients [19]. In a separate examination of triple-negative breast cancer, CD163 + TAMs emerged as an independent prognostic factor. It is essential to note that TAMs are dynamic cells, and their polarization into M1 or M2 phenotypes does not signify terminal differentiation. In response to the influence of various cytokines within the tumor microenvironment, TAMs exhibit a capacity for functional plasticity. They can transition from a pro-inflammatory phenotype to a suppressive one, and their polarization can also shift, with M1-type TAMs capable of converting into M0 or M2 subtypes. Currently, the differentiation of TAMs into subtypes that impede tumor progression represents a prominent area of interest in the field of cancer therapy.

### PD-L1 and macrophages

PD-L1, also known as B7-H1 or CD274, belongs to the cell surface glycoproteins of the B7 family. Under physiological conditions, PD-L1 is expressed in tissue cells and interacts with the programmed death receptor-1 (PD-1) on the surface of lymphocytes. This interaction serves to protect the body from damage resulting from an excessive inflammatory response, and it also plays a crucial role in autoimmune tolerance and the prevention and treatment of autoimmune diseases. When a tumor develops, tumor cells that exhibit high levels of PD-L1 expression hinder lymphocyte function and cytokine release, induce lymphocyte apoptosis, and thereby evade lymphocyte-mediated destruction. This immune evasion leads to the progression of the tumor [20]. In addition to tumor cells, TAMs also display elevated levels of PD-L1 expression and mediate immune regulation through various mechanisms. TAMs with high PD-L1 expression predominantly engage in immune regulation by binding to the PD-1 receptor on CD8+ T lymphocytes, eliciting the recruitment and activation of phosphatase SHP2. This results in the dephosphorylation of downstream protein kinases such as Syk and PI3K, subsequently down-regulating signaling pathways including mTOR, AKT, and ERK2. This downregulation inhibits the proliferation and survival of effector T cells, reduces the secretion of IFN-γ and TNF-α, negatively regulates T cell activity, and mediates T cell apoptosis. Consequently, this leads to a decline in the number of tumor-killing lymphocytes and diminishes the anti-tumor efficacy of the immune system [21].

Lim et al. [22] demonstrated that tumor necrosis factor-alpha (TNF-α) secreted by macrophages leads to increased PD-L1 expression in breast cancer through TNF-α-mediated NF-κB activation. Recent investigations into hepatocellular carcinoma [23] have revealed that osteopontin (OPN) facilitates the M2-like polarization of macrophages and induces PD-L1 expression in hepatocellular carcinoma by activating the colony-stimulating factor-1 (CSF1)/CSF1 receptor (CSF1R) pathway. This process amplifies the production of immunosuppressive cytokines and drives liver cancer cell metastasis by successfully evading Th1-dependent tumor elimination. Furthermore, analysis of clinical data from liver cancer patients supported a positive correlation between OPN, PD-L1, and TAMs infiltration. The foamy appearance of PD-L1+ TAMs is partially attributed to the accumulation of substantial uncleared phagocytic substances and lysosomes in the cytoplasm. Researchers speculated that PD-L1 might influence the phagocytosis capacity of TAMs. To validate this hypothesis, PD-L1+ TAMs and PD-L1- TAMs were categorized, and in vitro phagocytosis assays were conducted using GFP+ *Staphylococcus aureus* biologics. Comparatively, the phagocytic function of *Staphylococcus aureus* by PD-L1+ TAMs was impaired when contrasted with PD-L1-TAMs, signifying that PD-L1+ TAMs were under a state of phagocytic inhibition. This hypothesis has also been substantiated by in vivo studies. The expression of PD-L1 in TAMs adversely modulates their phagocytic capacity against tumor cells. It has been observed that PD-L1+ TAMs express F4/80 and CD11b at similar levels to PD-L1-TAMs. However, PD-L1+ TAMs exhibit a higher expression of the M2-related scavenger receptor CD206, increased CD11c, and reduced MHC II expression [24]. This observation suggests that PD-L1 can enhance the differentiation of macrophages into M2 phenotypes. Furthermore, studies with PD-L1 knockout mice revealed the development of severe peritonitis with prominent infiltration of M1 macrophages and an upregulation of pro-inflammatory molecules. This indicates that PD-L1 deficiency promotes the differentiation of macrophages towards the M1 phenotype and intensifies the enzyme-polysaccharides induced inflammatory response by enhancing STAT1/p-NF-κB p65 phosphorylation [25].

### CD47 and macrophages

CD47, also known as integrin-associated protein (IAP), is an immunoglobulin-like protein extensively expressed on cell membranes, encompassing both normal cells and various types of tumor cells, including leukemia, lymphoma, and a diverse array of solid tumors. CD47’s ligand is the signal regulatory protein alpha chain (SIRPα), primarily present on the surface of macrophages, dendritic cells, and nerve cells. The interaction between cell surface receptors and ligands governs cell migration, phagocytosis activity, immune homeostasis, and neuronal network regulation. CD47 on the surface of normal cells interacts with SIRP-α on macrophages’ surface, thereby inhibiting the phagocytosis of normal cells by macrophages. Various tumor cells suppress TAMs’ phagocytosis by elevating the expression of CD47 protein, ultimately enabling immune escape. Research has indicated that diminishing the expression of CD47 on tumor cells significantly enhances TAMs’ ability to eliminate tumor cells [26].

CD47 expression restricts macrophage activity and suppresses the non-specific immune system by binding to SIRPα. For example, decreased CD47 expression on the surface of red blood cells enhances erythrocyte phagocytosis by macrophages in the red pulp of the spleen, a critical pathogenic factor in hemolytic anemia [27]. Studies of malignancies such as leukemia, non-Hodgkin’s lymphoma, bladder cancer, and breast cancer have revealed elevated CD47 levels in tumor cells, with high CD47 expression correlating with poor clinical prognosis [28]. Cancer stem cells, characterized by high genomic instability and drug resistance, underlie tumor dissemination and metastasis. Several types of cancer stem cells also exhibit CD47 overexpression. Blocking the CD47-SIRPα pathway with anti-CD47 antibodies enhances phagocytosis of tumor cells. Published phase I clinical results show varying safety profiles for Hu5F9-G4 and TTI-621 [29]. All 16 patients treated with Hu5F9-G4 developed varying degrees of anemia, and some experienced hyperbilirubinemia, although no cases of low platelet count were reported. In contrast, in the group receiving 0.3 mg/kg TD-621, 4 out of 5 patients developed severe (Grade 3 and 4) thrombocytopenia, but all 11 patients maintained stable hemoglobin levels with no anemia. The critical difference in these outcomes may be attributed to the active utilization of CD47’s aggregation effect to mitigate adverse reactions related to red blood cells [30]. The fusion protein TTI-621 specifically recognizes aggregated CD47. It has been reported that the hemoshadotin skeleton restricts CD47 aggregation on the surface of human erythrocytes. Therefore, TTI-621 capitalizes on the challenge of aggregating erythrocyte CD47 to achieve tolerance to human erythrocytes, thus averting adverse reactions that could lead to anemia [31]. As mentioned earlier, preclinical models of anti-CD47 therapy may have overestimated efficacy, and the response to treatment can greatly vary among different tumor types. Consequently, real clinical outcomes might not directly align with the results from animal experiments, underscoring the need for careful patient selection based on CD47 expression and aggregation levels. Notably, ovarian cancer represents an ideal candidate for CD47 antibody therapy due to its unique anatomical structure and pathophysiological characteristics.

### MHC class I component β2-microglobulin /LILRb1 signal

Researchers have observed that even after inhibiting the expression of CD47, certain tumor cells can still evade macrophage phagocytosis. Another recognition mechanism between tumor cells and macrophages has been identified, involving the signaling molecule on the surface of tumor cells that shields them from macrophage phagocytosis, known as the major histocompatibility complex I (MHC I) class component β2-microglobulin [32]. Blocking or reducing the expression of this molecule can activate macrophages in vivo and enhance their phagocytic activity. This, in turn, leads to the elimination of tumor cells and a significant extension in the survival of tumor-bearing mice by up to 70%. Furthermore, when researchers knocked out leukocyte immunoglobulin-like receptor subfamily B1 (LILRB1) on the macrophage surface, which is recognized by MHC I, macrophages transition from promoting tumor growth to inhibiting it [33]. Current research indicates that the inhibitory LILRB1 protein often features a common clone, GHI/75, which, when combined with anti-CD47 monoclonal antibodies, significantly boosts macrophages’ ability to engulf and kill tumor cells. Importantly, the inhibition of LILRB1 does not harm normal tissue cells in vivo [34, 35].

### CD24-Siglec-10 signal transduction

CD24 exerts a crucial role in the immune system by regulating inflammatory responses and immune cell activity, contributing to the maintenance of immune balance. However, CD24 is also overexpressed in various cancers and is associated with cancer cell invasion, migration, and drug resistance. Its interaction within the tumor microenvironment can inhibit immune cell attacks, promoting cancer cell survival. Additionally, high CD24 expression may be linked to the formation of cancer stem cells, which are more resistant to treatment, making cancer more challenging to cure. Therefore, CD24 not only plays a regulatory role in normal immune function but also serves as a key player in cancer development, making it a potential target for cancer treatment and research. In addition to the previously mentioned “do not eat me” signal, researchers have identified additional potential escape signals during investigations into the strength and durability of responses to therapeutics like monoclonal antibodies. In studies involving breast and ovarian cancer, BARKAL et al. [36] discovered that CD24 serves as a prominent innate immune checkpoint and a promising target for tumor immunotherapy. Their research illustrated that CD24-expressing tumors facilitate immune evasion by interacting with the inhibitory receptor sialic acid-binding Ig-like lectin 10 (Siglec-10) expressed on TAMs. Subsequent investigations revealed that CD24 overexpression occurs in other types of tumors, not limited to breast and ovarian cancer, and that TAMs exhibit high levels of Siglec-10 [37]. Disrupting the interaction between CD24 and Siglec-10 using monoclonal antibodies or eliminating CD24 or Siglec-10 led to enhanced phagocytosis of CD24-expressing human tumors by TAMs. These findings introduce novel concepts to the field of tumor immunotherapy.

## Anti-tumor therapy targeting macrophages

In recent years, tumor immunotherapy has garnered significant attention and witnessed substantial advancements. Immunotherapy aims to restore the equilibrium between the immune system and tumors by modulating the body’s immune defense mechanisms, reprogramming immune cells, or utilizing diverse immunomodulatory agents. Notably, both CAR-T cell therapy and PD-1/PD-L1 inhibition have demonstrated remarkable clinical efficacy. Given their pivotal role in the tumor microenvironment, macrophages have emerged as promising targets for the development of tumor immunotherapeutic agents, capitalizing on their intrinsic characteristics. In the following sections, we will provide a comprehensive overview of various tumor immunotherapy strategies that specifically target macrophages, along with their prospective applications. An overview of relevant drugs is presented in Table 2 for reference.

**Table 2:** Tumor immunotherapy strategies and their application prospects

Category	Substance	Target site	Mechanisms of action
Inhibitor	Zoledronic acid	CCL2	Suppress the expression of CCL2 [38]
	Gefitinib	CCL5	Decrease the secretion of CCL5 [39]
	PLX3397	CSF1R	Inhibit the expression of CSF1R [40]
	GW2580	CSF1	Inhibit the expression of CSF1 [41]
	Wortmannin	PI3K	Decrease serum cytokine levels by inhibiting PI3K [42]
Monoclonal antibody or blocker	HAC	PD-LI	Block human PD-LI [43]
	BMS-936558	PD-1	Block the interaction between PD-1 and PD-L1 [44]
	Hu5F9-G4	CD47	Block CD47 that induces tumor-cell phagocytosis [45]
	KWAR23	SIRPa	Combine with tumor-opsonizing antibodies to aug-ment neutrophils and TAMs antitumor activity [46]
Biological response modifier	GHI/75	LILRB 1	Block the MHC /LILRB1 signaling way [47]
	Trabectedin	Macrophages	Block the immunosuppressive effect [48]
	Immunomodulator linemode	Macrophages	Block the activity of macrophages in tumor angio-genesis [49]
	DNMTi AZA (5-Azacytidine)	Macrophages	Regulate of macrophages polarization [50]
	DFMO (a-Difluoromethylornithine)	Macrophages	Regulate of macrophages polarization [51]
	DNTs (dual-inhibitor-loaded nanoparticles)	M2 macrophages	Make M2 macrophages repolarize to active MI macrophages and inhibit CSF IR and SHP-2 [52]

### The CCL2 and CCL5

Activated macrophages, monocytes, and dendritic cells exhibit heightened secretion of CCL2, also known as monocyte chemoattractant protein-1 (MCP-1), in response to stimulation by proinflammatory factors like IL-8 and TNF-α. M2-type TAMs collaborate with CCL2 to facilitate tumor progression by attracting macrophages. Due to its role in promoting cancer, CCL2 has been recognized as a potential target for impeding TAM recruitment to tumors [53]. Recent studies have shown that zoledronic acid can downregulate the expression of CCL2, resulting in a reduction in macrophage recruitment and exhibiting antitumor effects [54]. Furthermore, increased secretion of CCL5 in certain instances can also attract TAMs by binding to CCR2 on the surface of monocytes [55].

### Colony-stimulating factor-1 (CSF-1)

Tumor cells secrete CSF-1, which binds to CSF1R on macrophages, initiating downstream pathways to recruit and polarize TAMs. Therefore, the primary approach to targeting TAMs involves inhibiting TAM polarization, recruitment, and cytokine secretion by blocking the CSF1/CSF1R pathway. A study [56] demonstrated that the tyrosine kinase inhibitor PLX3397 substantially reduces CSF1R expression in a BrAFV00E-driven mouse melanoma model. This drug, with its CSF1R inhibition, has been employed in the treatment of patients with glioblastoma and breast cancer. Research has indicated that the proportion of M2 TAMs decreases significantly with the reduction of TAMs. Similarly, in MMTV neu transgenic mice, GW2580 (a specific CSF1 inhibitor) led to a considerable reduction in TAM infiltration in tumor tissue [57]. It is generally accepted that the loss of the CSF1/CSF1R signal specifically depletes M2 TAMs while having minimal impact on M1 TAMs [58].

### Signal transduction blockade of related kinases

IL-10 enhances tumor growth and metastasis by upregulating CIP2A expression via the PI3K signaling pathway. It has been demonstrated [59] that the phosphorylation of cAMP response element-binding protein (CREB) can also regulate IL-10 secretion by E6-positive lung cancer cells through PI3K pathways. The feedback loop involving IL-10, CIP2A, and CREB phosphorylation may impact tumor progression. Targeted therapy can interrupt this signaling pathway using specific inhibitors like wortmannin or LY294002 (PI3K inhibitor). Wortmannin, a frequently employed cell biology agent, has been used to impede DNA damage repair, receptor-mediated endocytosis, and cell proliferation [60]. Wortmannin has been shown to lower serum levels of certain cytokines by inhibiting PI3K/Akt pathway activation, consequently hindering tumor invasion [61]. In a recent study, Halaby et al. [62] discovered that serine-threonine kinases can influence the maturation and polarization of macrophages and myeloid-derived suppressor cells by regulating the translation of non-repressor 2 into the transcription factor CREB-2/activating transcription factor 4. Targeting ATF4 with small interfering RNA effectively disrupted GCN2-related signaling pathways, leading to the inhibition of tumor growth. These findings suggest that blocking GCN2-related signaling pathways can enhance anti-tumor immunity.

### PD-1/PD-L1 signal transduction blockade

In one study, immunodeficient mice were treated with either a PD-L1 blocker (HAC, a small molecule protein that blocks human PD-L1) or a PD-1 blocker (anti-mouse PD-1 antibody BMS-936558). The results demonstrated that both mouse and human TAMs expressed high levels of PD-1, and the PD-1 levels increased progressively with tumor development [39]. Inhibition of PD-1/PD-L1 led to an enhancement in the phagocytic activity of TAMs, resulting in tumor cell destruction. Furthermore, in macrophage-mediated immunotherapy, PD-1/PD-L1 may interact with CD47 mab, and combination therapy showed a higher survival rate compared to monotherapy [63]. PD-1 mab has received FDA approval for clinical use due to its remarkable efficacy in treating certain advanced malignancies, even though it is effective in only a small subset of cancer patients [64].

### Macrophage polarization regulation

In recent years, with the profound advancements in the molecular biology of liver cancer, molecular targeted therapies have achieved significant breakthroughs in hepatocellular carcinoma (HCC) treatment. Therapeutic strategies targeting macrophages within the HCC microenvironment aim to induce the conversion of M2 macrophages into M1 macrophages and to counteract immune suppression. Trabectedin, a macrophage-targeted drug initially designed for the treatment of soft tissue sarcomas [65], is a marine bioactive extract known for its specific cytotoxicity against macrophages. Other potential drugs, such as the immunomodulator LineMode, act to inhibit macrophage activity in tumor angiogenesis. Furthermore, the use of a CCL2 antibody has the potential to reduce macrophage aggregation and may be explored as a treatment option. C-fms, a CSF receptor that plays a pivotal role in regulating macrophage function, is an emerging focus of clinical studies. Combinations of drugs aimed at modulating TAM polarization could influence the interaction between C-FMS and other immune cells, thereby altering macrophage phenotypes and reshaping the microenvironment to limit the prevalence of M2-type TAMs [66].

The inhibition of TAM polarization through combined drug therapy holds significant promise in clinical applications. A recent study by Travers [67] demonstrated that the combination of DNMTI 5-azacytidine (AZA) and α-difluoromethylornithine (DFMO) significantly enhanced survival rates and reduced tumor burden in mice with ovarian cancer. When applied to an ovarian cancer mouse model with normal immune function, this combined drug treatment substantially extended the survival of tumor-bearing mice. Notably, the combination therapy led to a significant reduction in M2 TAMs, concomitant with a marked increase in M1 TAMs. These findings underscore the potential of combined treatment in influencing macrophage polarization within the tumor microenvironment, recruiting M1 macrophages, and prolonging the survival of individuals with tumors. Furthermore, a recent study by RAMESH [68] introduced self-assembled dual inhibitor-loaded nanoparticles (DNTs) designed to target M2-type TAMs and transform them into active M1-type TAMs. This approach also involved the simultaneous inhibition of the CSF1R and SHP-2 signaling pathways. The findings from this study present an innovative avenue for anti-tumor therapy focused on targeting macrophages, and DNTs exhibit promising potential for clinical translation as a personalized therapeutic option.

## Conclusion

In conclusion, a comprehensive understanding and effective utilization of the intricate interactions within the tumor immune microenvironment hold the potential to enhance the efficacy of tumor immunotherapy and address the challenges posed by the low response rates observed in immunotherapy. As precision medicine gains prominence, the focus of anti-tumor treatments has shifted towards precise targeted therapy. In light of the growing significance of anti-tumor immunity, there has been a surge in research efforts aimed at overcoming longstanding challenges in traditional tumor therapy. However, it is essential to acknowledge that recent years have seen limited progress in the realm of adaptive immunity. Previous studies have illustrated the multifaceted impact of macrophages on tumor cells, elevating this field to a prominent position within immunotherapy research. Researchers have identified specific cytokines secreted or modified by macrophages, demonstrating their potential in combatting tumor cells. The relentless commitment of scholars in the medical field to conduct in-depth investigations has led to the discovery of diverse mechanisms governing the recognition of TAMs. Various targeted therapies, including the utilization of monoclonal antibodies, inhibitors, gene modifications, and the adoptive transfer of immune cells, are subjects of in-depth investigation. This underscores the considerable promise of macrophages in the realm of targeted tumor therapy. Presently, there exists a plethora of therapeutic approaches; however, their technological maturity is still evolving, and clinical trials are relatively scarce. Consequently, numerous unidentified molecular mechanisms may wield significant influence over the regulation of tumor growth and progression. Some prospective targets warrant further extensive research and attention. A more profound examination of the intricate interplay between macrophages and tumor cells is imperative.

## Data Availability

The data presented in this study are available upon request from the corresponding author.

## References

[1] Vanpouille-Box C, Lhuillier C, Bezu L (2017). Trial watch: immune checkpoint blockers for cancer therapy (J/OL). OncoImmunology.

[2] Lequeux A, Noman MZ, Xiao M (2021). Targeting HIF-1alpha transcriptional activity drives cytotoxic immune effector cells into melanoma and improves combination immunotherapy. Oncogene.

[3] Li XY, Wenes M, Romero P (2019). Navigating metabolic pathways to enhance antitumour immunity and immunotherapy. Nat Rev Clin Oncol.

[4] Xia LZ, Oyang L, Lin JG (2021). The cancer metabolic reprogramming and immune response(J/OL). Mol Cancer.

[5] Leone RD, Powell JD (2020). Metabolism of immune cells in cancer. Nat Rev Cancer.

[6] Boroughs LK, Deberardinis RJ (2015). Metabolic pathways promoting cancer cell survival and growth. Nat Cell Biol.

[7] Hurley HJ, Dewald H, Rothkopf ZS (2021). Frontline science: AMPK regulates metabolic reprogramming necessary for interferon production in human plasmacytoid dendritic cells. J Leukoc Biol.

[8] Shi H, Yan KK, Ding L (2020). Network approaches for dissecting the immune system(J/OL). iScience.

[9] Li J, Wang Q, Xia G (2023). Recent advances in targeted drug delivery strategy for enhancing oncotherapy. Pharmaceutics.

[10] Clara JA, Monge C, Yang Y (2020). Targeting signalling pathways and the immune microenvironment of cancer stem cells—a clinical update. Nat Rev Clin Oncol.

[11] Mohammadi A, Blesso CN, Barreto GE (2019). Macrophage plasticity, polarization and function in response to curcumin, a diet-derived polyphenol, as an immunomodulatory agent. J Nutr Biochem.

[12] Gordon S, Plüddemann A (2017). Tissue macrophages: heterogeneity and functions. BMC Biol.

[13] Schridde A, Bain CC, Mayer JU (2017). Tissue-specific differentiation of colonic macrophages requires TGFβ receptor-mediated signaling. Mucosal Immunol.

[14] Zhou J, Zhang S, Guo C. Crosstalk between macrophages and natural killer cells in the tumor microenvironment. Int Immunopharmacol. 2021;101(Pt B):108374. 10.1016/j.intimp.2021.108374 34824036

[15] Vitale I, Manic G, Coussens LM (2019). Macrophages and metabolism in the tumor microenvironment. Cell Metab.

[16] Wang H, Yung MMH, Ngan HYS (2021). The impact of the tumor microenvironment on macrophage polarization in cancer metastatic progression. Int J Mol Sci.

[17] Hasong J, Ilseon HJ, Sun HK (2019). Tumor-associated macrophages as potential prognostic biomarkers of invasive breast Cancer. J Breast Cancer..

[18] Jamiyan T, Kuroda H, Yamaguchi R (2020). CD68- and CD163-positive tumor-associated macrophages in triple negative cancer of the breast. Virchows Arch.

[19] Liang YL, Lin CN, Tsai HF (2021). Omental macrophagic “crown-like structures” are associated with poor prognosis in advanced-stage serous ovarian cancer. Curr Oncol.

[20] Mezzadra R, Sun C, Jae LT (2017). Identification of CMTM6 and CMTM4 as PD- L1 protein regulators. Nature.

[21] Li CW, Lim SO, Xia W (2016). Glycosylation and stabilization of programmed death ligand- 1 suppresses T- cell activity. Nat Commun.

[22] Lim SO, Li CW, Xia W (2016). Deubiquitination and stabilization of PD-L1 by CSN5. Cancer Cell.

[23] Zhu Y, Yang J, Xu D (2019). Disruption of tumour-associated macrophage trafficking by the osteopontin-induced colony-stimulating factor-1 signalling sensitises hepatocellular carcinoma to anti-PD-L1 blockade. Gut.

[24] Gordon SR, Maute RL, Dulken BW (2017). PD-1 expression by tumour-associated macrophages inhibits phagocytosis and tumour immunity. Nature.

[25] Chen W, Wang J, Jia L (2016). Attenuation of the programmed cell death-1 pathway increases the M1 polarization of macrophages induced by zymosan. Cell Death Dis.

[26] Severino PF, Silva M, Carrascal M (2017). Expression of sialyl-Tn sugar antigen in bladder cancer cells affects response to Bacillus Calmette Guerin (BCG) and to oxidative damage. Oncotarget.

[27] Brierley CK, Staves J, Roberts C (2019). The effects of monoclonal anti-CD47 on RBCs, compatibility testing, and transfusion requirements in refractory acute myeloid leukemia. Transfusion.

[28] Moran I, Grootveld AK, Nguyen A (2019). Subcapsular sinus macrophages: the seat of innate and adaptive memory in murine lymph nodes. Trends Immunol.

[29] Arkhypov I, Lasser S, Petrova V (2020). Myeloid cell modulation by tumor-derived extracellular vesicles. Int J Mol Sci.

[30] Bai YP, Yu H, Wang K (2017). Research status and application prospect of anti-CD47 targeted therapy. Chin J Cancer Clin.

[31] Cantrell MS, Wall JD, Pu X (2022). Expression and Puri-fication of a cleavable recombinant fortilin from Escherichia coli for structure activity studies. Protein Expr Purif.

[32] Hanahan D (2022). Hallmarks of Cancer: new dimensions. Cancer Discov.

[33] Chen Q, Li Y, Gao W (2021). Exosome-mediated crosstalk between tumor and tumor-associated macrophages. Front Mol Biosci.

[34] Xu J, Lin H, Wu G (2021). IL-6/STAT3 is a promising therapeutic target for hepatocellular carcinoma. Front Oncol.

[35] Li W, Wang F, Guo R (2022). Targeting macrophages in hematological malignancies:recent advances and future directions. J Hematol Oncol.

[36] Barkal AA, Brewer RE, Markovic M (2019). CD24 signalling through macrophage Siglec-10 is a target for cancer immunotherapy. Nature.

[37] Shen W, Shi P, Dong Q (2023). Discovery of a novel dual-targeting D-peptide to block CD24/Siglec-10 and PD-1/PD-L1 interaction and synergize with radiotherapy for cancer immunotherapy. J ImmunoTher Cancer.

[38] George CN, Canuas-Landero V, Theodoulou E (2020). Oestrogen and zoledronic acid driven changes to the bone and immune environments: potential mechanisms underlying the differential anti-tumour effects of zoledronic acid in pre-and post-menopausal conditions. J Bone Oncol.

[39] Zhou J, Tang Z, Gao S (2020). Tumor-associated macrophages: recent insights and therapies. Front Oncol.

[40] Fujiwara T, Yakoub MA, Chandler A (2021). CSF1/CSF1R signaling inhibitor pexidartinib (PLX3397) reprograms tumor-associated macrophages and stimulates T-cell infiltration in the sarcoma microenvironment. Mol Cancer Ther.

[41] Ahmad SF, Duncan WC, Campbell LL (2020). Targeting colony stimulating factor-1 receptor signalling to treat ectopic pregnancy. Sci Rep.

[42] Lin WH, Jiang WP, Chen CC (2022). Renoprotective effect of pediococcus acidilactici gka4 on cisplatin-induced acute kidney injury by mitigating inflammation and oxidative stress and regulating the MAPK, AMPK/SIRT1/NF-κB, and PI3K/AKT pathways. Nutrients.

[43] Kamiya D, Takenaka-Ninagawa N, Motoike S (2022). Induction of functional xeno-free MSCs from human iPSCs via a neural crest cell lineage. NPJ Regen Med.

[44] Shin J, Phelan PJ, Gjoerup O (2021). Characterization of a single chain variable fragment of nivolumab that targets PD-1 and blocks PD-L1 binding. Protein Expr Purif.

[45] Sallman D, Donnellan W, Asch A (2019). S878 the first-in-class anti-CD47 antibody Hu5F9-G4 is active and well tolerated alone or in combination with azacitidine in AML and MDS patients: initial phase 1b results. HemaSphere.

[46] Li C, Xu X, Wei S, et al. Tumor-associated macrophages: potential therapeutic strategies and future prospects in cancer. J ImmunoTher Cancer. 2021;9(1):e001341. 10.1136/jitc-2020-001341 PMC8728363 33504575

[47] Chen H, Chen Y, Deng M, et al. Antagonistic anti-LILRB1 monoclonal antibody regulates antitumor functions of natural killer cells. J ImmunoTher Cancer. 2020;8(2):e000515. 10.1136/jitc-2019-000515 PMC7418854 32771992

[48] Allavena P, Belgiovine C, Digifico E (2022). Effects of the anti-tumor agents trabectedin and lurbinectedin on immune cells of the tumor microenvironment. Front Oncol.

[49] Putra WE, Agusinta AK, Ali Ashar MSA (2023). Immunomodulatory and ameliorative effect of Citrus Limon extract on DMBA-induced breast Cancer in mouse. Karbala International Journal of Modern Science.

[50] Pant R, Kabeer SW, Sharma S, et al. Pharmacological inhibition of DNMT1 restores macrophage autophagy and M2 polarization in western diet-induced nonalcoholic fatty liver disease. J Biol Chem. 2023;299(6):104779. 10.1016/j.jbc.2023.104779 PMC10248527 37142224

[51] Peters F, Becker-Pauly C (2019). Role of meprin metalloproteases in metastasis and tumor microenvironment. Cancer Metastasis Rev.

[52] Lou JWH (2023). Applications of porphyrin nanoparticles in enhancing Cancer immunotherapies.

[53] Tu M, Klein L, Espinet E (2021). TNF-alpha-producing macrophages determine subtype identity and prognosis via AP1 enhancer reprogramming in pancreatic cancer. Nat Cancer.

[54] Cheng J, Yang Z, Ge XY (2022). Autonomous sensing of the insulin peptide by an olfactory G protein-coupled receptor modulates glucose metabolism. Cell Metab.

[55] Oh JH, Hur W, Li N, Jo SJ (2022). Effects of the epidermal growth factor receptor inhibitor, gefitinib, on lipid and hyaluronic acid synthesis in cultured HaCaT keratinocytes. Exp Dermatol.

[56] Zhao Y, Jun W, Yang A (2021). Effect of spinal cord colony-stimulating factor 1 on morphine analgesia tolerance in rats. J Bengbu Med College.

[57] Cassetta L, Pollard JW (2018). Targeting macrophages: therapeutic approaches in cancer. Nat Rev Drug Discov.

[58] Malfitano AM, Pisanti S, Napolitano F (2020). Tumor-associated macrophage status in cancer treatment. Cancers.

[59] Byrnes K, Blessinger S, Bailey NT (2022). Therapeutic regulation of autophagy in hepatic metabolism. Acta Pharm Sin B.

[60] Bader JE, Voss K, Rathmell JC (2020). Targeting metabolism to improve the tumor microenvironment for Cancer immunotherapy. Mol Cell.

[61] Boovarahan SR, Kurian GA (2021). Preconditioning the rat heart with 5-azacytidine attenuates myocardial ischemia/reperfusion injury via PI3K/GSK3β and mitochondrial KATP signaling axis. Biochem Mol Toxicol.

[62] Halaby MJ, Hezaveh K, Lamorte S (2019). GCN2 drives macrophage and MDSC function and immunosuppression in the tumor microenvironment. Sci Immunol.

[63] Sugiura D, Okazaki IM, Maeda TK (2022). PD-1 agonism by anti-CD80 inhibits T cell activation and alleviates autoimmunity. Nat Immunol.

[64] Xu S, Wang X, Yang Y, et al. LSD1 silencing contributes to enhanced efficacy of anti-CD47/PD-L1 immunotherapy in cervical cancer. Cell Death Dis. 2021;12(4):282. 10.1038/s41419-021-03556-4 PMC7969769 33731702

[65] Serdà PC, Terés R, Sebio A (2022). Single-center experience with Trabectedin for the treatment of non-L-sarcomas. Adv Ther.

[66] Banerjee S, Halder K, Ghosh S (2015). The combination of a novel immunomodulator with a regulatory T cell suppressing antibody (DTA-1) regress advanced stage B16F10 solid tumor by repolarizing tumor associated macrophages in situ. Oncoimmunology.

[67] Travers M, Brown SM, Dunworth M (2019). DFMO and 5-azacytidine increase M1 macrophages in the tumor microenvironment of murine ovarian cancer. Cancer Res.

[68] Ramesh A, Kumar S, Nandi D (2019). CSF1R- and SHP2-inhibitor-loaded nanoparticles enhance cytotoxic activity and phagocytosis in tumor-associated macrophages. Adv Mater.

